# Menstrual hygiene – A salient hazard in rural schools: A case of Masvingo district of Zimbabwe

**DOI:** 10.4102/jamba.v8i2.204

**Published:** 2016-01-13

**Authors:** Everson Ndlovu, Ednah Bhala

**Affiliations:** 1 Institute of Development Studies, National University of Science and Technology, Zimbabwe; 2 Maranatha Orphans Care Trust, Maphisa, Zimbabwe

## Abstract

Active participation of the girl child in development is hampered by Menstrual Hygiene Management (MHM) challenges. MHM is an important gender issue and a critical component in holistic human development. It affects about 25% of the global population aged between 15 and 49 years. Water, sanitation and hygiene (WASH) interventions in schools have not prioritised MHM, thus exposing girls and the entire school community to health related hazards. The study explored knowledge, attitudes and community practices, and investigated the impact of religious and cultural beliefs on MHM and how they impact on the girl child in Masvingo district. The survey was largely qualitative and employed methodologies of document analysis, Focus Group Discussions (FGD) and structured interviews. Participants included four churches, 13 NGOs, eight government departments and 40 women. Findings revealed deeply embedded power relations, a culture of silence around MHM, noninvolvement of men in MHM issues, limited availability in terms of information, and a girl unfriendly infrastructure, and limited access to menstrual hygiene products due to poverty and poor management and disposal practices. Resultant effects ranged from poor class participation, lack of concentration and constrained interactions with peers and teachers, low self-esteem, anxiety and the general feeling of being discriminated against. Results confirmed the need for increased awareness initiatives on MHM in a bid to tackle inherent religious and cultural beliefs that are a barrier to effective holistic implementation of WASH interventions that empower women and girls. Lobbying government to provide an appropriate policy framework, education and training, construction of girl friendly sanitary facilities, exploring and capitalisation of local production of Reusable Menstrual Pads (RUMPS), more research targeting children living with disabilities, those living in refugee and makeshift camps and Orphans and Vulnerable Children (OVC), are some of the recommendations coming out of the study.

## Introduction

Despite numerous efforts to promote girl child education in Zimbabwe, Menstrual Hygiene Management (MHM) has not been given adequate attention in both schools and within communities. MHM is an important gender issue and a critical component in holistic human development and yet in Zimbabwe, the issue remains clouded in cultural taboos, restrictions and unhygienic practices that further compound the health related hazards for women and girls particularly in poor rural communities. Masvingo Province located in the low veld of the country where rainfall is minimal and erratic has a large rural poor population. Most parts of the province, therefore, are generally unfit for agriculture, apart from cattle ranching. Inadequate water sources contribute to poor sanitation and hygiene, the key determinants in effective MHM. Culture and traditions are strongly embedded in the people’s way of life making them so conservative that changing behaviour is a mammoth task; thus, cultural taboos expose women and girls to increasing vulnerabilities. Lack of sensitivity on MHM can create psychological and/or emotional scars (i.e. traumatic stress disorders) that derail girls in terms of realising their full potential, thus contributing to their failure to achieve MDG 2. National water, sanitation and hygiene (WASH) interventions have been negatively affected by limited evidence. As a result, there has been an inclination to treat girls and boys as homogenous in WASH programming for schools and communities. In addition, gaps and inadequate capacity in terms of rights based programming have been identified as other contributing factors to poor performance by girls in school. The study aims at highlighting prevalent menstrual hygiene practices, attitudes, religious and cultural beliefs as they impact on the girl child. It is envisaged that the findings will inform both duty bearers and rights holders to design relevant and rights based programmes that will improve the wellbeing of women and girls.

## Research objectives

The intention of the study was to explore community knowledge levels, their attitudes and general community practices in the area of Menstrual Hygiene Management and to establish their impact on the girl child in Zimbabwe. These variables were interrogated within the context of community culture and religious belief systems which this study further explored and documented. Menstrual Hygiene Management related challenges in schools especially were identified and recommendations were made to promote sustainable girl friendly interventions in Zimbabwean schools. The aims were to:

- Explore general community knowledge, attitudes and practices on MHM and how they impact on the girl child in Zimbabwe.
- Document cultural and religious belief systems that impact on MHM in Zimbabwe.
- Identify MHM related challenges faced by girls in Zimbabwean schools.
- Recommend sustainable girl friendly MHM interventions.

### Literature review

The discourse on menstruation is cloaked in secrecy and negativity; in many countries it is associated with cultural and religious taboos, and is therefore completely neglected (Jain 2013). Inadequate MHM in communities perpetuates inequalities between genders that already exist to hamper the empowerment of women and girls; 20% of rural primary school girls, that menstruate, do not attend school whilst they are on their menses and the explanation that they give to their parents and teachers is that they are sick with a headache, stomach ache or some other ailment (Mtigwe *et al*. 2014). If girls are missing education because of menstruation, this reduces their future career prospects (Mutunzi 2013). There has not been considerable research on MHM and interventions implemented; various actors in terms of sexual and reproductive health have been silent on the subject. The reaction to menstruation depends upon awareness and knowledge about the subject. The manner in which a girl learns about menstruation and its associated changes may have an impact on her response to the event of menarche.

The availability of WASH facilities, their functionality, adequacy, utilisation, state, and maintenance leave a lot to be desired and expose school communities to health related hazards (SNV 2012). There is no standardisation in the curriculum resulting in either different contextual assimilations of the content or possibility of some WASH topics being completely neglected. Teachers are generally inadequately equipped to handle the mainstreaming of MHM (Ministry of Primary and Secondary Education 2012). Most schools are unable to provide appropriate sanitary ware for girls and pain killers for those girls who experience dysmenorrheal resulting in some girls choosing to absent themselves from school during menses to avoid the associated loss of self-esteem, discomfort and stigma from their schoolmates. Some girls hide in the bush during the entire period of menses and walk back home after school hours, fooling parents or guardians into believing that they were attending school (Mutunzi 2013). Most rural schools do not have water sources within 500 m of the school grounds as provided for in the Sphere Minimum Standards (2011). Such a compromised scenario negatively impacts on menstrual hygiene in terms of washing blood spoiled hands, pads, underwear and uniforms (Ministry of Primary and Secondary Education 2012).

Stigma around menstruation and menstrual hygiene is a violation of girls’ right to human dignity, right to nondiscrimination, equality, bodily integrity, health, privacy and the right to freedom from inhumane and degrading treatment (Patkar 2015). The designation of male teachers as MHM counsellors is likely to fail, taking into account that discussing menstruation with men is a cultural taboo, let alone by a lay male teacher who is likely to be assigned to take up the role because there would be no female teacher available. Hence, there is no doubt that such arrangements create a barrier to gaining knowledge and compromises the delivery of effective education on this subject. Instead, such an approach can result in unintended outcomes whereby teachers end up abusing the girls or having affairs with them (Piper 2011). Shangwa (2011) points out that most rural schools are characterised by a lack of water for washing hands or spoiled uniforms and privacy for changing pads in the design of ‘Blair’ toilets that have no doors and cannot prevent other girls entering whilst the other girl is still changing her sanitary pad. The majority of girls are not happy with the current state of the Blair toilets as it provides no privacy during menses. Menstrual related absence from school results in a girl losing out on 528 days of schooling across the years that a girl should be in school. Consequently, these results in lost opportunities for these girls and the beginning of differences affecting their economic and social standing compared to boys (Shangwa 2011).

Old pieces of cloth, old panties, leaves, socks, tissue, old newspapers and cotton wool were cited as common materials used by girls in their communities (Mtigwe *et al*. 2014). Shangwa (2011) posits that the majority of girls in Zimbabwe use inappropriate materials that compromise their health. A feasibility study on the establishment of sanitary products manufacturing units in Gokwe, Mt Darwin and Chipinge districts in Zimbabwe, portrays a similar picture even in districts endowed with cotton (Ministry of Women Affairs, Gender and Community Development n.d). Unaffordability clearly stands out to be a hindrance or barrier in the use of conventional sanitary pads for many girls and women who coincidentally belong to low income households that also are characteristic of the majority of households in Zimbabwe.

## Research methodology

### Research approach

In 2012, the Ministry of Primary and Secondary Education conducted a survey in 211 schools in the Masvingo district to establish the nature and extent of water sanitation and hygiene challenges. The survey targeted the school community (teachers and students) as respondents. In 2014, SNV-Zimbabwe wanted to implement a WASH in schools programme in Masvingo, and needed comprehensive baseline information to justify the need for the project. The authors, Everson Ndlovu and Ednah Bhala, conducted the study on behalf of SNV but this time targeting critical stakeholders in Masvingo. School girls were not part of this study because their views had already been captured by the 2012 study by the Ministry of Primary and Secondary Education. The Ministry of Primary and Secondary Education Survey (2012) in WASH programmes in schools in Msvingo captured the views of the school community only (teachers and students). Schools alone would not provide comprehensive data to allow for informed interventions on MHM. This study brings in other critical voices and issues from the major stakeholders in the WASH sector (NGOs, CBOs and FBOs) and government agencies, the disabled persons and women’s groups. A survey research design was adopted for this study. It was largely qualitative in nature and solicited for views, perceptions, beliefs and knowledge levels on MHM. Major stakeholders (public sector and NGOs) were targeted and these constituted key informants. These were both public sector departments, churches and NGOs operating in Masvingo district and having offices in Masvingo town with a few located in the outskirts of the city. Participants to the study also dealt with school going girls directly or indirectly through School Development Committees or traditional leaders.

### Sampling procedure

A purposive sampling procedure was employed in picking respondents to the study. All stakeholders working with schools and directly or indirectly interacting with the girl child and members of the WASH cluster in Masvingo were targeted. To this end, eight public sector departments, four churches and 13 NGOs constituted key informants. In total, 30 key informants (15 male and 15 female) respondents out of an envisaged 40 participated in the study through structured questionnaire. These were selected on the basis of availability and willingness to discuss MHM issues; 40 additional women participated through Focus Group Discussions and were drawn from women’s organisations that worked directly and indirectly with the girl child in school.

### Face-to-face in-depth interviews with key informants

Secondary stakeholder views on MHM intervention and/or modelling of intervention, challenges that may be encountered, gaps and possible linkages and lessons learnt from their previous experiences were solicited through face-to-face interviews where possible, otherwise the tool was designed to allow stakeholders to fill in and write additional notes in an atmosphere where they would be free to express themselves on the subject of MHM. Thirty out of the expected 40 individual questionnaires (15 males and 15 females) were administered over a four-day data collection period. Some individuals were either not keen to discuss the subject of MHM or needed clearance from their heads of departments who were not available during the study period. Nonetheless, organisations and government departments that responded were seen as a major stakeholder grouping. The research team used structured questionnaires and administered the questionnaires to solicit for data from the key informants. It took the team approximately 30 min to go through each questionnaire. The male researcher focussed mainly on male respondents as men were not comfortable answering questions on menstruation from a woman.

### Focus Group Discussions

Focus Group Discussions (FGDs) with women drawn from community structures like WASH Committees, Village Health Workers, Child Protection Committees, Community Home-Based Care Givers, School Development Committees, and the Women’s Coalition of Zimbabwe, Gender Focal Persons, District Aids Committees and some women from church groupings. These groups were critical because they understood the challenges women and girls face during their menses and some were already doing work on advocacy issues for women and girls. Four FGDs were conducted by a female researcher with a total of 40 women. Women felt at ease discussing such sensitive issues with a female researcher. Data on MHM knowledge, awareness, attitudes, practices, beliefs, expectations, impressions and/or anticipated challenges were collected through FGDs. The qualitative assessment utilising FGDs sought to identity gaps and to make recommendations for future modelling of the planned MHM intervention in Masvingo district and Zimbabwe in general. A FGD guide was used to give direction to the flow of the discussions. A significant amount of probing ensured that more salient issues were brought to the surface and that barriers to MHM communication were minimised as a result. Data were collected over 4 days including data from Key Informant Interviews. A gender sensitive approach was undertaken in order to facilitate the analysis of the two gender views as far as possible with respect to MHM.

### Observations

Observation was also employed as a tool in the study. Some reactions and attitudes amongst respondents presented around the discussions of menstruation were captured by observing reactions and issues that had been raised in the FGDs and one-to-one interviews as being best practices or challenging cases. Observations of situations and activities helped to confirm what people said in interviews (triangulation). As the team conducted interviews, observations of nonverbal communication of the respondents were made. Men were shy to answer certain questions and would not look at the researcher directly, an indication that culturally they would not discuss certain elements on the nature of menstruation.

In terms of their level of education, 77% of the respondents had a university undergraduate degree; 47% held senior management positions; 23% were in middle management; and 17% were field officers. Half of the participants worked directly with school girls whilst others worked with girls indirectly through School Development Committees, hospitals and community engagements.

In terms of interaction, 77% of the respondents interacted with the girl child in their daily routine although the bulk of the respondents confessed they did not address MHM issues with the girl child. This was an indication of the neglect in terms of MHM and the subsequent lack of support for the girl child. Thirteen per cent never interacted with the girl child; if they did, it was indirect through School Development Committees (SDCs), ward councillors or traditional leaders. Traditional leaders and government officials reported that they hardly talked about issues to do with girl children at school as the majority of them were men; 10% of the respondents indicated they interacted with the girl child at times and the majority of these were NGOs. Whilst the respondents identified MHM as a gap in WASH programmes, they did not have the capacity and resources to incorporate MHM into their interventions.

## Data analysis

The research data gathered through various tools were classified into thematic areas for analysis purposes. The data were processed both manually and using SPSS. Graphical presentations were presented for value addition and ease of interpretation of some of the results. Data were collated, analysed and synthesised. Running themes or patterns were identified, interpreted and explained accordingly.

## Ethical considerations

Authority to conduct a research study was sought with the responsible authorities in the district. The necessary protocol was observed, with the District Administrator being the first port of call. Such an approach facilitated easy of movement in both the city and Masvingo district. Informed verbal consent was obtained from all participants for all data collection participants (FGDs and key informant stakeholders). All interviewees were assured of confidentiality in terms of the information that they provided. Individual interviews were conducted in private settings.

## Findings and discussions

### Perceptions

Respondents presented varying perceptions about MHM in schools in Masvingo; 24% of the respondents described MHM as a critical area in Masvingo that needed addressing, yet they also consider the MHM programme as one that has been mismanaged and not prioritised and not emphasised, silent, privatised, stigmatised, and thereby leaving the girl child with little support, uncomfortable and with a significant challenge during their menstruation. The majority of women in FGDs indicated that menstruation is a private issue which is rarely discussed as a result of religious and cultural beliefs around it. Men were said not to be involved in MHM and the responsibility lay squarely on women’s shoulders. Respondents indicated that most communities are fraught with gender inequalities where women and girls have no decision making power, women have limited access to and control over resources, and these issues become a barrier in effective management of menstrual issues, as men tend to prioritise other matters that either concerned themselves or the broader family.

Girls start menstruating as early as 8 years of age. This was attributed to the kind of food they eat with some singling out eggs as the main cause. Girls confide in their mothers or female relatives about menstruation. However, the relationship that the girl had with the mother is significant, because if the mother was not approachable, the girl would rather consult her friends. If menstruation started at school, girls would generally be more comfortable confiding in female teachers than male teachers because they are socialised to believe that menstruation is not to be discussed with men.

### Religious and cultural beliefs and their impact on the girl child

MHM is a taboo topic in most communities in Masvingo, in line with what Shangwa (2011) postulated. Menstruating girls and women were not allowed to touch animals, to get close to water points, not to prepare or touch food that others would eat, and were not to shake hands with men when greeting them. Women and girls were excluded from religious rituals because they were considered unclean during menses. Men and boys were not supposed to know that the girl was menstruating; she was not supposed to cook for them because that would make them ‘weak’. Ironically, some of the practices like excluding the girls from church activities and household chores were tantamount to announcing that the girl was menstruating yet it was supposed to be kept a secret. If one had painful period pains, they would not have children. Women and girls were not allowed to fetch water in a stream, or bath with running water in a stream. They were also not allowed to pass in the midst of livestock as it would cause the animals to become barren. On the first menses, a particular person was assigned to clean the blood; if this ritual is not followed that person will not bear any children.

There was also a belief that only traditional healers could cure dysmenorrheal. Blood stained materials were disposed of or dried privately to avoid witchcraft. Some of the myths promote unhygienic practices like drying pants under the bed, yet it should be dried in the sun to kill any germs or bacteria. This invariably calls for an urgent address by all the stakeholders to entrench correct menstrual perceptions and to enable proper hygiene practices amongst school girls (Thark *et al*. 2011). The information given to the girls makes them more anxious and affected the performance at school, for example, the issue that if one had painful period pains they would not have children; the same observation was made by Mtigwe *et al*. (2014). It is therefore imperative to engage traditional and religious leaders as the custodians and gatekeepers of the beliefs that hinder girls from receiving adequate information and guidance on MHM. Cultural and religious beliefs create insecurity and withdrawal by school girls, resulting in limited participation in class, extracurricular activities and peer interaction.

### Attitude of boys towards menstruating girls

**FIGURE 1:**
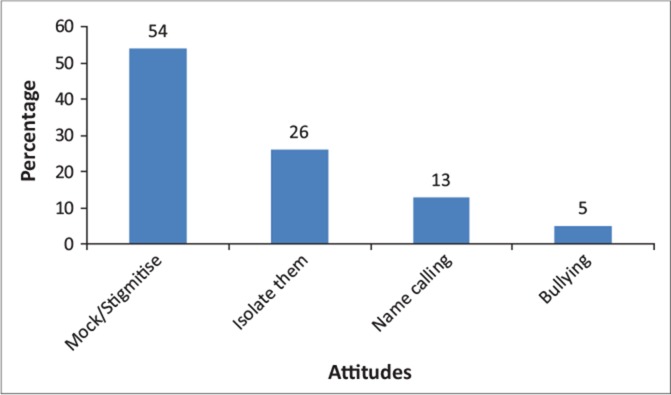
Attitudes of boys towards girls.

**FIGURE 2:**
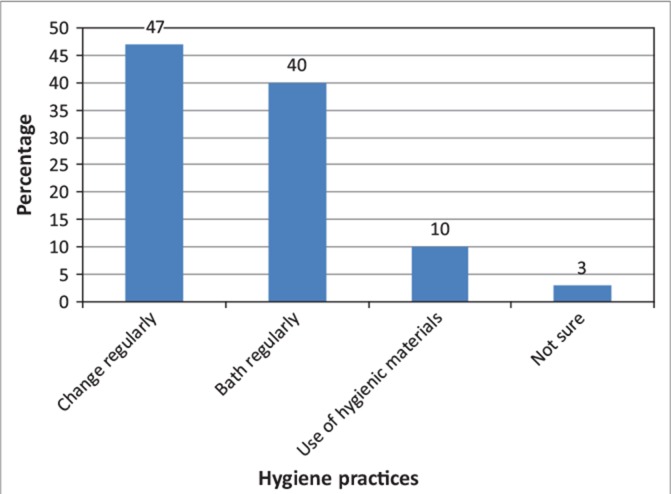
Hygiene practices by girls.

Varied negative attitudes were displayed by boys towards girls who are menstruating (Figure 1). These included boys mocking, stigmatising (54%) or isolating girls (26%), as well as bullying (5%) them and name calling (13%) as shown in Figure 2. The negative attitudes by boys could result in some girls eventually withdrawing from school, coupled with the physical discomfort due to menstruation. Such negative attitudes are heavily influenced by lack of appropriate information and more directly by cultural beliefs. These are boys that will grow into manhood with all these ingrained stereotypes that are likely to perpetuate such negativity if not managed from the onset. As long as boys continue with this kind of attitude, it will mean whatever mitigation strategies aimed at assisting the girl child will yield negative results, thus further exposing the girls to health risks.

### Hygienic practices

Figure 2 shows hygiene practices by women and girls; 47% respondents felt there was a need for girls to change sanitary ware regularly to mitigate health related hazards, 40% of the respondents cited bathing regularly as a priority, 10% indicated use of hygienic materials as vital whilst 3% were not sure of proper hygiene standards. Maintaining hygiene during menses is important for women’s well-being, mobility and dignity. Such an assertion was also made by Shangwa (2011). Practices like using hygienic materials, changing sanitary ware frequently, bathing and washing and proper disposal of the used materials whilst considered ideal, were far from being observed given the various challenges girls face. Girls would simply fold their used sanitary ware and rap it in a plastic bag for washing at home or for disposal elsewhere as culturally menstrual blood should never be seen. Such a practice exposes the girls and their families to health related risks.

### Barriers to hygienic practices

The inhibiting factors to girls observing hygienic practices mainly hinged around the issue of MHM being a private matter; this limits girls’ access to information and support that they could get at school and at home. In terms of the challenges experienced by respondents:

- 30% indicated water challenges in terms of sanitary facilities at school
- 35% indicated the majority of the girls could not afford sanitary ware as it was priced beyond their capacity
- 10% of the girls had no underwear which meant they would rather stay at home than face embarrassment at school
- 15% indicated they had access to proper disposal facilities
- 5% complained of a lack of reusable sanitary ware.

**FIGURE 3:**
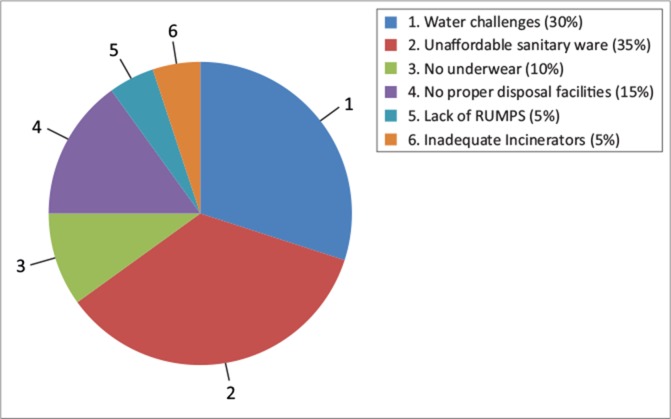
Barriers to good menstrual hygiene.

At school level, most of the sanitary facilities were reported as inappropriate to provide for the privacy that a girl needs if she was to change her pads and bath. Most toilets were said not to have hand washing facilities, had no doors and some of the facilities were poorly maintained (Muduma 2014). MHM was a challenge in most communities due to a shortage of water, limited information and disposal constraints (Figure 3).

### What do girls use and why?

#### Are the materials available and affordable?

**FIGURE 4:**
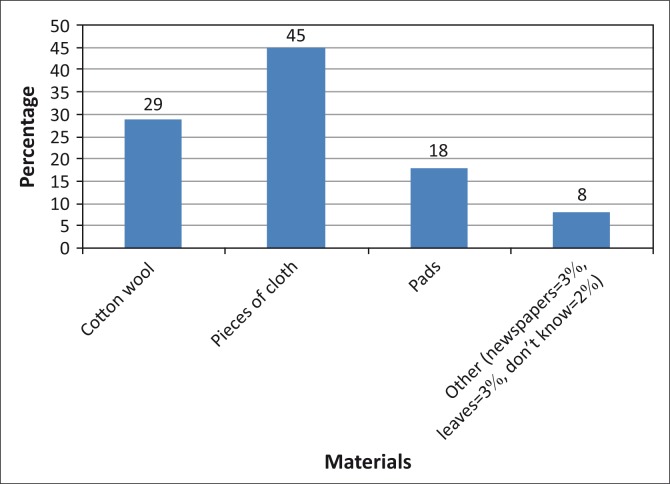
Materials used by girls.

Figure 4 shows materials used by girls during menses:

- 45% indicated girls used pieces of old clothes and rags
- 29% indicated that cotton wool was used
- 18% of the respondents used pads
- 3% of the respondents indicated that they use newspapers and leaves even though these caused discomfort, bruises and infections.

Once infected, their right to education was denied as they would have to be hospitalised. Cotton wool and pads were used by children from affluent families. Some girls were reported to be using pieces of cloth that they shared with their mothers due to poverty. Sanitary ware was said to be available in most communities but it was not affordable for the majority. In some instances, sanitary ware was not prioritised. Sanitary pads were the preferred method for MHM as girls felt more comfortable and confident when using them.

### Disposal of used materials

**FIGURE 5:**
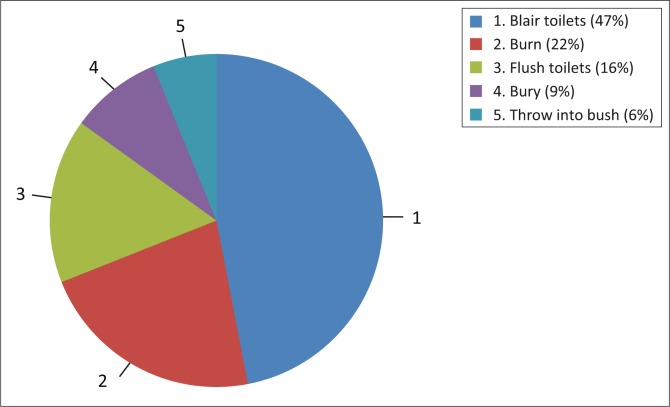
Methods of disposal.

Girls preferred disposing of pads, as opposed to washing and reusing them. Disposal methods included throwing used materials into the bush (6%), Blair toilets (47%), pits or latrines, and burying (9%) as shown in Figure 5. The disposal method was said to be determined by the belief systems of the family with some families believing that one’s blood should never be seen by others or the person would be bewitched. In such cases, they would rather burn (22%) the material used or wash it and dispose of it without any trace of blood.

### Sources of Menstrual Hygiene Management information

**FIGURE 6:**
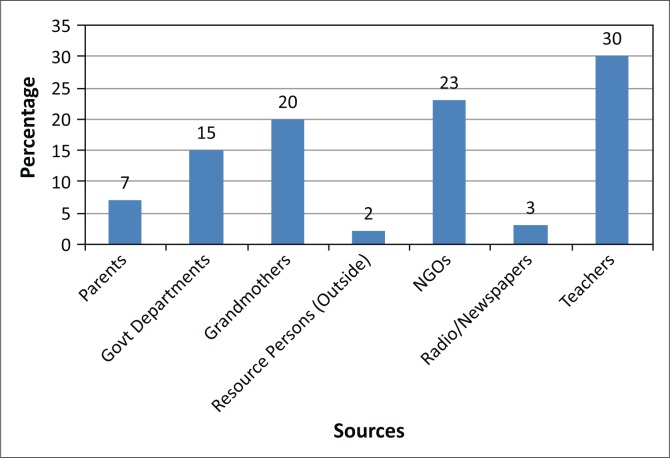
Sources of Menstrual Hygiene Management information.

In terms of available information, girls’ information sources are shown in Figure 6. Teachers emerged as the main sources of information (63%). Parents, grandparents, mothers, aunts and sisters (26%) followed by CBOs or NGOs or FBOs at (13%). Mothers or aunts were the ones most likely to buy pads for their daughters and nieces when they could afford them. Of particular note is the fact that none of the NGOs were considered to be sources of information. The health centres, even though they were accessible, were reported as not providing any MHM information. Respondents indicated that even though health facilities were accessible, some girls did not want to visit them for fear of stigmatisation and that they may be using family planning methods. The government departments (20%) that provided information included the Zimbabwe National Family Planning Council (ZFNPC), Social Welfare and Environmental Health Technicians.

### Impact of menstrual hygiene management on the overall education of the girl child

**FIGURE 7:**
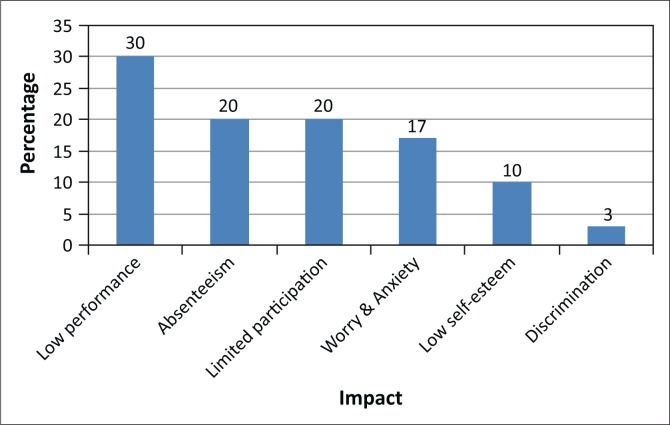
Menstrual Hygiene Management impact on girl education.

The general consensus was that menstruation had negative effects on the performance of the girl child in school (Figure 7); 57% felt that a lack of adequate support from family and school led to increased absenteeism, 30% indicated low performance, whilst 20% cited lack of concentration amongst girls. Seventeen per cent cited low participation in class and extracurricular activities for the girl child. Girls become anxious, have low self-esteem, felt discriminated against, and silently suffered; these factors had a negative bearing on their educational performance. Many schools do not support adolescent girls or female teachers in managing menstrual hygiene with dignity. The impact of poor MHM on the psychosocial wellbeing of girls (e.g. stress levels, fear and embarrassment, and social exclusion) affected their health ultimately contributing to poor results and inability to access opportunities thereafter. Inadequate WASH facilities make managing menstruation very difficult.

## Conclusion

MHM is a necessity and yet it is a challenging issue for the girl child. Girls need to receive information on practical ways of managing menses in a girl friendly environment and hygienic way. Formal menstruation education is grossly inadequate in most schools and communities in Masvingo although some education is provided informally in some schools, particularly private schools. Teachers and mothers were identified as the main sources of information on MHM. However, information on MHM given by mothers can sometimes be incomplete and incorrect, and is usually based on cultural myths, personal experiences and views, which may result in false perceptions and unsafe practices regarding menstruation. It is evident from the results that MHM cannot be tackled effectively without the active involvement of men. Men control resources and make decisions on how these are spent; their involvement would ensure that MHM is adequately catered for in families. Community and religious leaders also have an important role to play especially in the demystification of beliefs and practices that might have a negative bearing on the promotion of MHM. The gender unfriendly school culture and infrastructure, and the lack of adequate menstrual protection alternatives and/or clean, safe and private sanitation facilities for girls, undermine their right of privacy, health and education. A multisectoral approach in programming on MHM in the developing and promotion of positive attitudes towards MHM is critical if transformative change is to be realised.

### Recommendations

Government should enact appropriate policies, related legislation and guidelines for minimum standards on implementing, monitoring and evaluating MHM in schools and within the development context for Zimbabwe. Through the Ministry of Primary and Secondary Education, government should incorporate MHM into the school curriculum. NGOs, research institutes and other development players complimenting government efforts should identify simple design innovations to efficiently and effectively enhance MHM facilities in schools for girls and female teachers. These designs may include dustbins for disposal, incinerators and buckets of water inside latrines or toilet stalls, girl friendly toilets and doors with locks.

Communities, government, civil society and private sector should explore the sustainability of new sanitary protection products under development like Reusable Menstrual Pads (RUMPS), including how such products can be profitable and disposed of in an environmentally safe manner. Community women sewing clubs could be established, capacitated and linked with the market for distribution purposes. Fashion and fabrics in schools should consider RUMPs as items girls could specialise in. Engage the private sector to produce and distribute affordable and appropriate sanitary protection materials and disposal facilities. Various mobile phone operators could use their facilities to send impact MHM messages to cover a wider reach. There is need for further research and documentation of current MHM practices and the barriers girls face in various contexts especially for girls living with disabilities, OVC and those living in refugee camps to further strengthen the evidence base. In view of the vital role played by mothers, it is very important that the mother be armed with correct and appropriate information in terms of reproductive health, so that she can give this knowledge to her growing girl child. It is necessary to explore additional avenues and expand existing educational programmes targeted at girls and communities in order to break the silence around the subject and empower girls with adequate information and skills to successfully manage menses in school and at home. Programmes must strengthen the connections between the rights to water and sanitation and other rights, including health, education, food, work, land, freedom from violence, and the right to information (rights based programming). Health centres should be equipped to provide accurate and user-friendly information on the biological facts about menstruation, menstrual health and hygiene.
